# Penetrating canaloplasty in angle-closure glaucoma secondary to iridocorneal endothelial syndrome following multiple failed filtering surgeries: A case report

**DOI:** 10.1097/MD.0000000000032950

**Published:** 2023-02-22

**Authors:** Xuelian Tian, Juan Guo, Jinying Liao, Meng He, Yinwen Shi, Li Tang

**Affiliations:** a Department of Ophthalmology, West China Hospital, Sichuan University, Chengdu, Sichuan, China; b Department of Ophthalmology, The Third People’s Hospital of Chengdu, Chengdu, Sichuan, China.

**Keywords:** angle-closure glaucoma, iridocorneal endothelial syndrome, penetrating canaloplasty

## Abstract

**Rationale:**

Angle-closure glaucoma secondary to iridocorneal endothelial syndrome (ICE) is challenging to treat, especially in patients who have already undergone multiple surgical procedures. Long-term success is difficult to achieve with traditional filtration surgery again. This case report describes a novel nonbleb-dependent surgery for managing such a young patient.

**Patient concerns:**

A 30-year-old male with glaucoma secondary to ICE was referred to West China Hospital, Sichuan University for uncontrolled intraocular pressure following multiple failed filtering surgeries under maximum topical antiglaucoma medications in his right eye.

**Diagnoses:**

The patient was diagnosed with angle-closure glaucoma secondary to ICE in the right eye based on a series of ophthalmic examinations.

**Interventions:**

Penetrating canaloplasty was performed to manage glaucoma secondary to ICE in the right eye.

**Outcomes:**

The patient’s visual acuity improved, the intraocular pressure was reduced to 11 to 15 mm Hg through 30 months of follow-up, and no antiglaucoma medication or additional surgical procedures were needed.

**Lessons:**

Penetrating canaloplasty could be considered as an option for the treatment of refractory angle-closure glaucoma secondary to ICE with extensive angle adhesion.

## 1. Introduction

The iridocorneal endothelial syndrome (ICE) is a group of ophthalmic disorders characterized by structural and proliferative abnormalities of the corneal epithelium, affecting both men and women from young to middle age.^[1]^ Elevated intraocular pressure (IOP) may occur due to peripheral anterior synechiae. It has been reported that the prevalence of secondary glaucoma of ICE is 46% to 82%, and glaucoma is the main cause of vision loss in ICE patients.^[2]^ The treatment of glaucoma secondary to ICE remains a challenge despite several treatment methods. Medical treatment for lowering intraocular pressure is usually ineffective. According to previous research results, the failure rate of glaucoma filtering surgery (GFS) reached 67% at 5 years in ICE secondary glaucoma due to the continued growth of the endothelial membrane over the surgical fistula.^[3]^ The survival rates will be further reduced, and the problem becomes even more intractable when multiple GFS, including glaucoma drainage device (GDD) implantation, results in surgical failures.

In ICE, the outflow resistance occurs due to synechial contact between the iris and the trabecular meshwork (TM) and the posttrabecular outflow pathway may remain intact. Nonbleb-dependent surgery comes into consideration. Success might be achieved if a method was adopted to allow aqueous humor directly from the anterior chamber into schlemm canal (SC), not through the TM. Hu et al^[4]^ reported successful treatment of neovascular glaucoma coexisting angle-closure with penetrating canaloplasty surgery (PCP), which combines canaloplasty with trabeculectomy. Here, we report the surgical process and postoperative outcome of such a case involving angle-closure glaucoma secondary to ICE who underwent PCP surgery after multiple GSFs, including GDD failures.

## 2. Case presentation

A 30-year-old male presented with eye pain and blurred vision in the right eye for 4 years. Previously, he had been diagnosed with right eye glaucoma secondary to iridocorneal endothelial syndrome 4 years earlier with the highest IOP value measuring 48 mm Hg, and the maximum topical antiglaucoma medications were not adequate to control elevated IOP. Then, he underwent multiple GFSs, including 2 trabeculectomies and Ahmed glaucoma valve implantation, in the last 3 years, but unfortunately, all the operations failed. When the patient decided to be referred to our clinic, he had intraocular pressure over 40 mm Hg in the right eye despite the use of 4 topical antiglaucoma agents, and his vision worsened. The patient reported no history of trauma, systemic disease or previous eye disorders.

**Figure 1.**
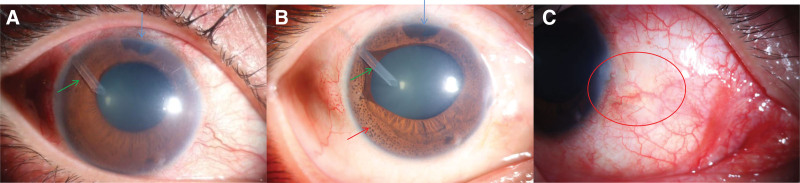
Photos of the anterior segment before (a) and after (b, c) surgery in the right eye. (a) Anterior segment photograph of the right eye before surgery showing corneal edema, pupil distortion, and multiple brown nodules. A glaucoma drainage tube (green arrow) and 2 iridotomies (blue arrow) can be seen in the right eye. (b) Photograph of the right eye after surgery showing clear cornea, aggravated pupil distortion and increased iris nodules (red arrow), but that cornea was still clear with normal IOP. (c) Slit lamp appearance of the right eye after surgery showing a flat bleb (red circle). IOP = intraocular pressure.

**Figure 2.**
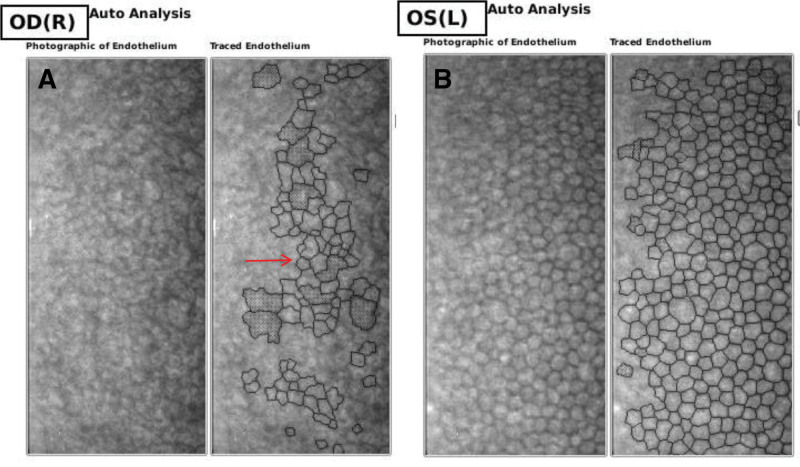
Report of corneal endothelioscopy in the right eye. Corneal endothelioscopy of the right eye (a) showing an abnormal endothelium morphology (red arrow) and less corneal endothelial cell density than the left eye (b).

**Figure 3.**
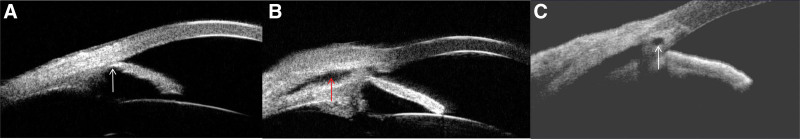
UBM and AS-OCT before (a) and after (b, c) surgery in the right eye. (a)UBM images of the right eye before surgery showing extensive peripheral anterior synechiae and closure of the drainage angle (white arrow). (b) UBM images of the right eye after surgery showing a deep scleral pool (red arrow) connected to the anterior chamber without obvious bleb. (c) AS-OCT of the right eye after surgery showing dilated SC (white arrow). SC = schlemm canal.

After admission, the relevant ophthalmic examinations were performed. The best corrected visual acuity was logMAR1.0 in the right eye (OD) and logMAR 0 in the left eye (OS). A tonometer revealed an IOP of 36.4 mm Hg in the right eye and 13.1 mm Hg in the left. Anterior segment exploration showed 3 flat and scarred blebs, corneal edema, pupil distortion, pigment eversion at the pupillary margin, iris atrophy and multiple brown nodules in his right eye. In addition, a tube was visible in the anterior chamber without corneal or lens touch, and 2 iridotomies after previous trabeculectomies could be seen in the upper part of the iris in the right eye (Fig. 1a). Corneal endothelioscopy revealed an abnormal endothelial morphology (Fig. 2), and corneal endothelial cell density could not be measured. In the same eye, extensive peripheral anterior synechiae and closure of the drainage angle can be revealed by ultrasound biomicroscopy (UBM) (Fig. 3a). Funduscopy indicated that the fundus of the right eye was not clear due to corneal edema, and the C/D was 0.8 with diffuse thinning of the neuro-retinal rim, suggesting optic nerve atrophy. Perimetry examination demonstrated inferior hemifields and superior nasal vision field loss. In his left eye, both the anterior segment and fundus examinations were unremarkable.

**Figure 4.**
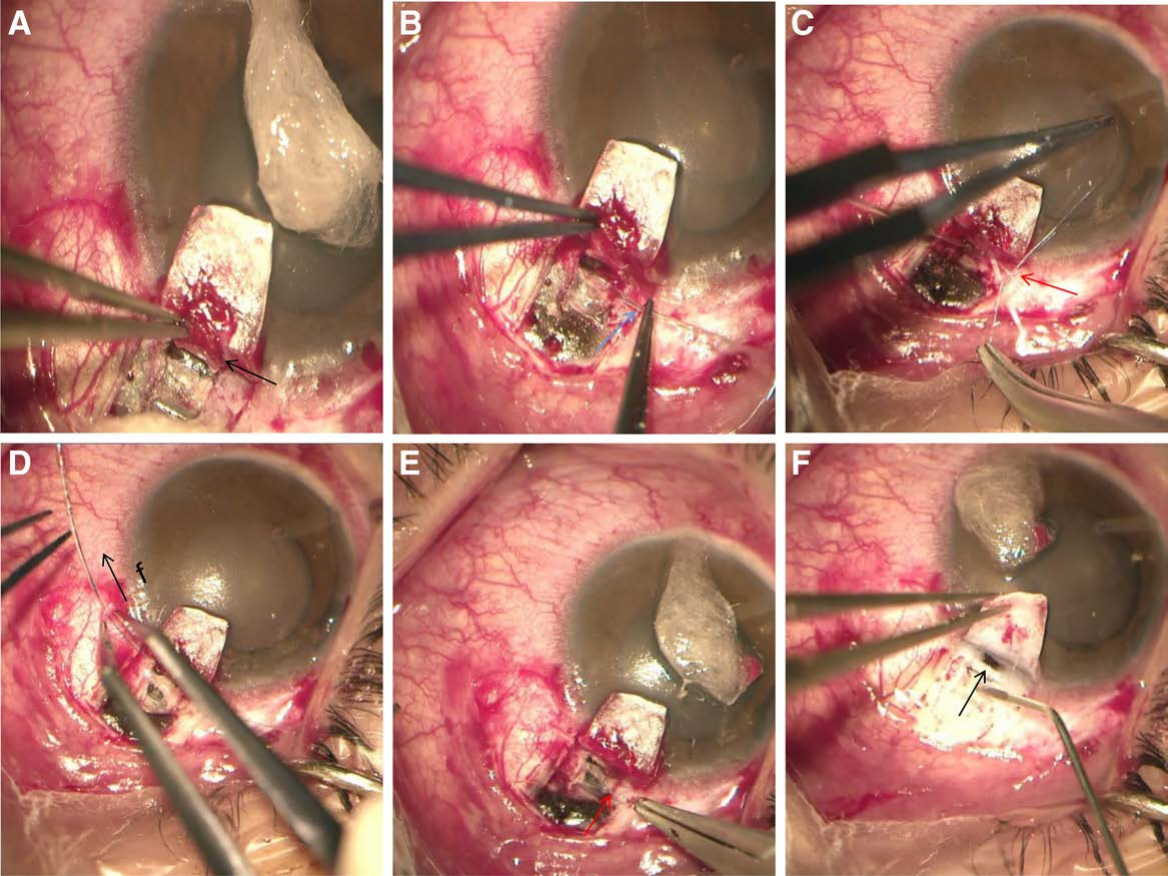
Photography of main steps in the operation. A deep scleral flap (black arrow) was dissected within the bed of the first scleral flap until the external wall of the Schlemms canal (a). The flexible microcatheter (blue arrow) guided by laser was inserted and leaded in the Schlemm canal to a circle (b). A single 10-0 prolene suture (red arrow) was tied to the tip (c). The microcatheter was withdrawn and leaded back through the canal in the opposite direction (black arrow showed the direction of force) and viscoelastics were injected into the canal at every 2 clock hours while the catheter was withdrawn (d). The prolene suture was retained in the Schlemm canal after ligation (red arrow) (e). A part of deep cornea and peripheral iris (black arrow) was removed to penetrate to anterior chamber (f).

After communicating with the patient about the possible surgical risks, a novel glaucoma surgery, PCP, which combines canaloplasty with trabeculectomy, was performed under local anesthesia. A nasal fornix-based conjunctival flap was created adjacent to the previous surgical site. A 4 × 4 mm superficial scleral flap was made, and a second deep scleral flap approximately 1.5 × 3 mm in size was dissected within the bed of the first scleral flap until the external wall of the Schlemms canal was opened. A laser-illuminating microcatheter (iTrack TM, Ellex iScience, Inc., Fremont, CA) was circumferentially inserted into Schlemm canal. As the previous surgical area was reached, the microcatheter encountered slight resistance but then passed through without further delay. A prolene suture with a diameter of 10.0 was tied and pulled back while a viscoelastic (Healon GV from Advanced Medical Optics, Santa Ana, CA) was injected for circumferential dilation of Schlemm canal after 360-degree catheterization. The deep scleral flap was removed, and a 2*2 mm deep corneal and scleral limbal tissue anterior to the SC was excised to penetrate the anterior chamber (AC). Then, peripheral irisectomy was performed to allow aqueous humor flow directly from the anterior chamber into the SC. Finally, the superficial sclera flap was tightly sutured with 10-0 prolene sutures, and the conjunctival flap was also sutured in a watertight manner to its original position. The main steps were shown in Figure 4a–f. This complicated surgery was uneventful. The eye was quiet postoperatively. On the first postoperative day, his visual acuity in the right eye was LogMAR 1.0, and his IOP was 13.5 mm Hg. A clear cornea with moderate depth AC was noted.

On the 10th day postsurgery, the patient IOP dropped to 8.7 mm Hg, but the AC remained stable. Anterior segment OCT showed mild ciliary detachment, so the patient was given prednisone 30 mg orally for 4 days, and ciliary body detachment was restored. The best corrected visual acuity improved to logMAR 0.5 2 months after the surgery, and the IOP remained stable for more than 2 years with no medication. After 1 month, 3 months, 6 months, 1 year, 2 years and 2.5years, the intraocular pressure was 12 mm Hg, 11 mm Hg, 14 mm Hg, 14.9 mm Hg, 13.6 mm Hg, and 17.7 mm Hg respectively. The cornea remained clear, although the pupil distortion was aggravated and the iris nodules increased (Fig. 1b). UBM and anterior segment OCT (AS-OCT) revealed a deep scleral pool connected to the anterior chamber (Fig. 3b) without obvious bleb (Fig. 1c) and dilated SC (Fig. 3c).

## 3. Discussion

Angle-closure glaucoma secondary to ICE is challenging to treat, especially in patients who have already undergone multiple surgical procedures.^[5]^ This case report describes the successful treatment with a new surgical method, PCP, for such a refractory glaucoma.

Filtering surgery in ICE-related glaucoma has a lower success rate than in other types of glaucoma.^[6]^ There is a possibility that the long-term failure of filtering surgery is caused by the proliferation of abnormal endothelial membranes over the TM and filtration site as well as the easy conjunctival scarring in young patients.^[7]^

Canaloplasty is a nonpenetrating glaucoma treatment method that dilates the SC with a microcatheter and a tension suture to restore the normal pathway for the outflow of aqueous fluids, but it is only suitable for open-angle glaucoma.^[8,9]^ Irreversible peripheral anterior synechiae obstructs the aqueous humor drains from the anterior chamber through the TM. PCP combines canaloplasty with trabeculectomy, creating a window connecting the AC with the SC directly.^[10,11]^ A small deep sclera helped keep the end of the incision of Schlemm canal open.Professor Liang and his team have reported that PCP demonstrated good safety and efficacy in eyes with primary angle-closure glaucoma, childhood glaucoma and corticosteroid-induced glaucoma.^[10,12–14]^Here we demonstrated its good safety and efficacy in eyes with angle-closure glaucoma secondary to ICE. PCP could be considered as an option for the treatment of a wide variety of refractory glaucoma.

Fortunately, although the patient had undergone 3 major surgeries for glaucoma, including 2 trabeculectomies, the entire 360 degrees Schlemm canal was passed by a microcatheter. The possible reason was that previous trabeculectomy only removed part of the deep corneoscleral tissue anterior to the SC, and the SC was intact. The insertion point of GDD was also kept away from SC. Trabeculectomy as a fistulization without touching the SC can provide a chance of success for later SC surgeries such as canaloplasty.

## 4. Conclusions

In this case, we provide a new nonbleb-dependent surgery for the treatment of angle-closure glaucoma secondary to ICE, and the surgery shows great efficacy for IOP reduction for at least 2.5 years without surgical complications. Therefore, we suggest that PCP could be utilized as an alternative treatment for angle-closure glaucoma secondary to ICE with extensive angle synechia. However, this paper is limited due to the single case report despite the relatively long follow-up time. Whether it can be considered the preferred treatment remains to be further confirmed by future more long-term outcomes of more cases and randomized controlled clinical trials.

## Author contributions

**Data curation:** Yinwen Shi.

**Investigation:** Juan Guo.

**Resources:** Jinying Liao, Meng He.

**Writing – original draft:** Xuelian Tian.

**Writing – review & editing**: Li Tang.
